# Ecology of Lactobacilli in the Oral Cavity: A Review of Literature

**DOI:** 10.2174/1874285800802010038

**Published:** 2008-04-29

**Authors:** C Badet, N.B Thebaud

**Affiliations:** Université Victor Segalen Bordeaux 2, UFR d’Odontologie, 16 cours de la Marne, 33000, Bordeaux, France

**Keywords:** Lactobacilli, oral cavity, caries, saliva

## Abstract

Lactobacilli appear in the oral cavity during the first years of a child’s life. Their presence depends on numerous factors such as the presence of ecological niches e.g. natural anfractuosities of the teeth.

A strong correlation has been established between the saliva *Lactobacillus* count and dental caries, the higher the DMF index, the higher the number of children harbouring a high *Lactobacillus *count.

Among children, the presence of lactobacilli in coronal caries is incontestable. Among adults, lactobacilli are found in root caries.

Since 1999, taxonomical revisions make it difficult to interpret the results obtained in the numerous previous studies carried out on the identification of oral lactobacilli, but whatever the sampling method or the identification technique, the carious site or the age of sampled subjects, most species belong to the *Lactobacillus casei* group.

This is important because if a specific correlation can be found between few species of lactobacilli and caries a better understanding of their properties could allow the development of new tools for prevention.

## INTRODUCTION

The oral cavity shelters a very numerous and various microbial flora. One major actor of this complex ecosystem is the dental plaque which develops naturally on oral tissues. This biofilm shows a very complex organization that remains relatively stable with time despite regular environmental changes [1-4]. When the equilibrium is compromised and when an imbalance appears among the indigenous bacteria, pathologies such as dental caries or periodontitis could occur [5].

Historically, lactobacilli were the first microorganisms implicated in dental caries development [6].

They appear during the first years of a child’s life, and are present in high numbers in saliva, on the dorsum of the tongue, mucous membranes, the hard palate, in dental plaque and, in fewer numbers, on tooth surfaces [7, 8]. The presence of lactobacilli in the oral cavity depends on numerous factors such as the presence of ecological niches e.g. natural anfractuosities of the teeth [9], partly erupted third molars or orthodontic devices [10-14].

During the last fifteen years, the *Lactobacillus *genus has been subjected to numerous taxonomical changes and includes at present more than 80 species [15], some of which having been found in the oral cavity. The use of molecular biology tools has cast doubt over the conventional classification. The taxonomy of lactobacilli is not easy because a lot of different Gram + bacilli are grouped together under this name (GC % from 32 to 53%). The analysis of ARNr 16S sequences of various species shows that they belong to three phylogenetically separate groups, apart from their morphological and physiological characteristics [16].

These taxonomical revisions make it difficult to interpret the results obtained in the numerous previous studies carried out on the identification of oral lactobacilli and the huge range of methods and results of these works justifies this review.

On the other hand, despite these disparate results, the salivary *Lactobacillus *count is used in the caries prediction tests. These current tests being not much specific, it seems to be of great interest to carry out a review of the knowledge about oral lactobacilli before the elaboration of new prediction tests.

### 1. Lactobacilli in Saliva

#### 1-a Salivary Lactobacilli in Sound Subjects

Saliva is a link between the different tissues and structures of the oral cavity. Its analysis reveals the oral microbiological characteristics. This principle is put into practice with the «caries detection test». A correlation also exists between *Lactobacillus* rates in dental plaque and in saliva [17]. If bacteria from the genus *Lactobacillus* represent 0.1% of the total salivary flora, a critical concentration of 10^5^ CFU/ml of saliva is necessary for the detection of lactobacilli on the surface of enamel [18].

Lactobacilli absent from the oral cavity of newborns, appear during the first year of the life. Mc Carthy *et al*. [19]observed the presence of this species in 50% of newborns during their first year with a rate from 200 to 30000 bact/sample.

In children without caries, the rate of salivary lactobacilli varied among the different studies.

Carlsson *et al*. [20] considered that lactobacilli became regularly present in 50% of children and only since the age of 2. Later, Köhler *et al*. [21], indicated that 40% of a population of 3-year old children harboured lactobacilli in rates varying from 2.10^3^ to 4.10^4 ^CFU/ml of saliva.

For older children (from 6 to 16 years old), this rate is slightly bigger (54.6%) [22].

On the other hand, other authors reported the presence of lactobacilli in 100% of sampled children [23, 24]. One factor that could influence the rate of salivary lactobacilli during childhood is the carbohydrate intake [25-27].

In young adults, their presence is not unconditional and it depends on appropriate ecological niches. Pits and fissures or partially erupted third molars provide a retentive environment favourable to the growth of these microorganisms [11, 28].

Other studies have shown a significative correlation between smoking habits and salivary rates of lactobacilli [29, 30].

In old subjects, the lactobacilli rate tends to increase again [31, 32]. Higher counts have been found in subjects harbouring removable prostheses [33] and in subjects consuming a lot of medicine [34, 35]. For Saotome *et al.* the measurement of the salivary lactobacilli level may be useful for the determination of oral health in elderly individuals[36].

Most of the studies were only quantitative. However, it seems to be interesting to identify the lactobacilli species present in the oral cavity of sound subjects in order to make appropriate comparisons with species found in saliva, dental plaque or dentin samples of subjects with caries.

Even if the multiplicity of methods makes it difficult to identify to the species level the lactobacilli found in saliva of children and adults without caries, we can notice a predominance of the *casei* group (Table **1**) [13, 37-39].

#### 1-b Salivary Lactobacilli and Carious Prediction

Evaluation of the carious risk is a long-standing concern whose aim is to target people who need intensive prevention and thus, improve the efficiency of public health programs.

The *Lactobacillus* count represents the number of lactobacilli present in 1ml of saliva (CFU/ml). It is used to determine the efficiency of dietetic measures or to evaluate the carious risk. It is used alone or associated with other parameters [40, 41]. For Granath *et al*. [42], it is a better criterion than the saliva rate of *Streptococus mutans*. A strong correlation has been established between the *Lactobacillus* count and caries [43-49],the higher the DMF index, the higher the number of children harbouring a high *Lactobacillus *count[39, 50].

Some studies have also been carried out in adults in order to investigate the link salivary lactobacilli / root caries. Results have shown a strong correlation between the* Lactobacillus *count and the presence of this kind of carious lesion [34, 51-53].Sullivan *et al.* [54] estimated that the presence of streptococci or lactobacilli in dental plaque is not a better indicator of carious activity than their count in saliva. This result is confirmed by the study of Motisuki *et al*. who showed the significance of the sampling method[55].

Therefore, the link *Lactobacillus* count/ carious decay is unquestionable. However, the relationship between the *Lactobacillus* count and the carious activity (i.e. the number of new caries appearing during a fixed period) and thus the determination of carious risk is much more questionable.

The study of van Palenstein *et al*.[56] seems to support the above theoretical considerations regarding the limited potential of bacterial counts as a caries predictor. On the other hand, a low level of *Lactobacillus* count seems to indicate, with a good probability, a low carious activity[57-61]. The clinical significance of the *Lactobacillus* count is also more reliable when applied to huge samples than to one person[62]. This test also has an interesting didactic application [63].

As in studies among sound subjects, the precise identification of lactobacilli present in saliva of a patient with caries has been difficult when biochemical methods have been used. With the advent of molecular tools, this identification has become more achievable. It is of some importance to know if a link exists between some species and caries, because it will be helpful for a better understanding of the natural reservoir of lactobacilli and, perhaps, it will allow the development of new tools for the prevention of dental decay[64].

The present results show again the predominance* of *the* L .casei* group [24, 65] (Table **2**).

#### 1-c Influence of Some General Pathologies on Salivary Lactobacilli

Some authors have investigated the influence of some general pathologies on the salivary lactobacilli rate. A correlation between diseases implicated in mouth dryness and the *Lactobacillus* count has been established. Antilla *et al*. [66] have demonstrated the association between the salivary *Lactobacillus* count and the symptoms of nervous breakdown. It therefore suggests an increased risk of dental diseasesamong depressed subjects, which should be recognized in theirtreatment. A probable factor in the correlation lactobacilli/nervous breakdown could be diet, as depressive subjects eat a lot of sweet products. Another explanation linked to a decrease in the immunological system may also be a contributing factor.

High counts of salivary lactobacilli have been related to hyposalivation or xerostomia[67, 68] . Bardow *et al*. described the relationships between the rate of tooth demineralisation and medication intake, subjective feeling of dry mouth, saliva flow, saliva composition and the salivary level of lactobacilli in adults aged from 44 to 84 years. They showed that subjects with low unstimulated flow rates (≤0.10ml/mn) had around 20 times more lactobacilli per ml of saliva than the subjects who had normal flow rates. This increase is greater in patients subjected to radiotherapy[69-72].

### 2. lactobacilli in Dental Plaque 

It is difficult, from the literature, to have an exact idea of the relationship between lactobacilli and dental plaque whether on the quantitative level or on the qualitative level. In effect, the numerous studies were carried out on different populations (age, DMF index, fluoride intake). Moreover, the sampling methods were also very varied. However, some results can be highlighted.

Some authors have noticed an increase in the rate of lactobacilli before the onset of carious lesions: this rate changes from 1% to 4 to 5 % [73, 74].

Even if lactobacilli have been reported to be less detectable in the dental plaque than in saliva [55, 65, 75], quantitative studies performed in children gave interesting results.

In plaque covering sound surfaces, various rates of lactobacilli have been found in the different studies. Van Houte *et al*. observed in a teenager population the presence of lactobacilli in about 50% of the 2 samples; Sigurjons *et al*.found lactobacilli in 62% of the subjects of the experiment whereas Babaahmady *et al*. identified Lactobacilli in 21% of their samples [76-78].

On the other hand, a positive correlation between the frequency of isolation of lactobacilli and the presence of white spot lesions was underscored. Beighton *et al*. carried out a survey in young children (3 to 4 years old); lactobacilli were isolated in 54% of children with caries and in 7% of children without caries. Matee *et al*. isolated lactobacilli from the dental plaque of children harbouring rampant caries and of children without caries. The number of lactobacilli was 100 fold higher in samples of children with caries [79, 80].

The studies performed in adult populations confirm an increase of the lactobacilli rate in dental plaque according to the presence of root caries [32, 53, 81, 82].

Qualitative studies confirmed the predominance of group *casei *species in dental plaque sampled on carious lesions. In children, *L. casei* is predominant, but, in early childhood caries, *L. fermentum* is the most frequently found species. The latter is also more frequently found in dental plaque samples in adults [20, 65, 83-87] (Table **3**).

As adherence is one major factor in the formation of dental plaque, some authors have investigated *in vitro *the correlation between the presence of lactobacilli in dental plaque and their capacity to coaggregate with other species.

Wilcox *et al*.[88] have demonstrated that of the 7 species of lactobacilli studied, only two were capable of coaggregation and the coaggregation was restricted to streptococci. *Lactobacillus salivarius* strains (2/4) coaggregated with *Streptococcus salivarius*, *Streptococcus gordonii*, *Streptococcus crista* and tufted *Streptococcus sanguis II* strains. *Lactobacillus fermentum* (2/3) coaggregated with *S. gordonii* and *S. sanguis*. The coaggregation between *L. salivarius* and *S. salivarius*, *S. gordonii* or tufted *S. sanguis II* strains was mediated by a protein on the surface of the lactobacilli and was not inhibited by lactose. The coaggregation between *L. fermentum *and the streptococci was mediated by protein on the surface of the streptococci and was inhibited by lactose.

Filoche *et al*. [89] have shown that biofilm formation by the lactobacilli in mono-culture was poor. In coculture with *Actinomyces* species the amount of *L. rhamnosus* increased 7-20 times and *L. plantarum* 4-7 times compared to its mono-culture biofilm*. S. mutans* also promoted substantial biofilm growth of lactobacilli but *V. parvula* had no effect. The authors conclude that these *Actinomyces *species promoted growth of key *Lactobacillus* species in a biofilm, as did *S. mutans* to a lesser extent, and that the ability of individual bacteria to form mono-culture biofilms is not necessarily an indicator of their survival and pathogenic potential in a complex multispecies biofilm community.

### 3. Lactobacilli on Mucous Surfaces 

Lactobacilli can also be present in great number on the surfaces of the tongue and the mucous[8, 73] . Michalek *et al.* [90] have shown that *Lactobacillus casei* selectively colonize soft tissues of the tongue in gnobiotic rats. Ahumada *et al*. [91] characterized lactobacilli from the tongue surface and the gums in children harbouring dental decay. 31 different species were identified; 78% came from the tongue and 22% from gums. The predominant strictly homofermentative species is *L. delbrueckii*; *L. coryneformis* and *L. plantarum* are the two predominant facultative heterofermentatives and *L. brevis* the dominant strictly heterofermentative. The same authors [85], using the same techniques also identified lactobacilli isolated from the tongue surface and gums in sound children. 42.3% of the lactobacilli came from the tongue and 11.8% from the gums. *L. fermentum* and *L. plantarum *are predominant on the tongue; *L. rhamnosus* is predominant on the gum. Ahrne *et al*. [92] used molecular biology tools in order to identify lactobacilli isolated from the surface of the tongue, in sound adults. *L.plantarum* was predominant (45%), *L.rhamnosus* (24%), *L.casei* 14%), *L. salivarius* (10%), *L. acidopilus* (7%) *L.oris* (2%), *L. fermentum* (2%).

The presence of these species on oral mucous could constitute a reservoir.

### 4. Lactobacilli in Carious Lesions

The microbial populations involved in dental caries are known to be highly complex and variable and have not yet been fully identified, although key organisms are generally recognized to be associated with disease progression.

Miller (1890) first demonstrated the bacterial invasion of dentinal tubules of both carious and non-carious dentine and reported that the tubuli micro flora contained rods. According to Owen[6], the association lactobacilli-dentinal caries was reported by Goadby in 1899*.* However, it was not until the late 1950s that experimental evidence clearly established the fundamental role of these bacteria in dental caries and in pulp and periapical disease[93]. They are now considered secondary invaders rather than initiators of the caries process[28].

The identification of lactobacilli species found in carious tissues has not been systematic. Some authors have only made a simple quantitative analysis of lactobacilli rates [83, 94-98].

Among children, the presence of lactobacilli in coronal caries is incontestable. However, they are found in less quantity than **Streptococcus mutans** and they are not found in incipient caries [84, 99-101]. The presence of these micro-organisms is also dependant on the size of the cavity: they are more numerous in medium and large cavities[102].

Among adults, lactobacilli are found in root caries [103, 104] but also in deep dentinal caries associated with pulpitis. Hahn *et al*. [105] have suggested the presence of two types of carious lesions: high level lactobacilli lesions and low level lactobacilli lesions. In high level lactobacilli lesions, the progression of these bacteria from necrotic superficial dentine to the deep level is very varied.

In van Strijp *et al’s* study [106] both sound and completely demineralized dentine were placed together in the partial prothesis of 8 individuals to test whether the type of substrate influenced the composition of the bacterial flora. The results showed that lactobacilli in the dentine specimens, was positively correlated to the lesion depth. (10.2 ± 13.9 %).

The authors, according to the work of McGrady *et al*. [107],**suggested an explanation for this observation: the presence of lactobacilli could be correlated to their affinity for dentinal collagen (90 % of the organic phase), which remained intact despite the demineralization.

Numerous authors, using different techniques, have identified species of lactobacilli found in carious tissues [65, 86, 87, 105, 108-114]. Whatever the sampling method or the identification technique, the carious site or the age of sampled subjects, most species belong to the *Lactobacillus casei* group (Tables **4** and **5**).

These studies have also revealed the variability of isolation frequency of lactobacilli among the carious lesions.

### CARIOGENIC PROPERTIES OF ORAL LACTOBACILLI

Even if dental decay cannot be considered as a nutritional illness, it results from an imbalance from daily carbohydrate consumption, because the cariogenic properties of bacteria is linked to sucrose metabolism which is implicated in two pathogenic properties, adherence and acid production.

Although the adhesive properties of lactobacilli are not noteworthy, it has been shown that they were able to adhere to various cell cultures and that the mechanisms of adhesion involved a certain level of specificity.

Lactobacilli cell surfaces have an S layer; this protein layer has a crystalline structure and is responsible for the surface hydrophobicity. The bacteria are able to adapt their surface hydrophobicity to environmental changes (pH, ionic strength). However, it has been demonstrated that strains with an S layer do not adhere better than strains without an S layer [115].

Some authors have studied the hydrophobicity of strains of lactobacilli isolated from dental plaque, tongue, gum and saliva in sound subjects and in patients with caries [86, 116]. A great number of lactobacilli isolated from the tongue, the dental plaque and the saliva showed hydrophobic properties from moderate to high. *L. acidophilus* and* paracasei* are the most hydrophobic species. *L.rhamnosus* and *L.plantarum* show a high level of hydrophobicity in dental plaque, gums but they also show a low one when isolated from saliva and tongue.

The production of exopolysaccharides is a key factor in the adherence of dental biofilm. Among some lactobacilli species, it has been studied extensively in the food industry, however there are fewer studies concerning oral lactobacilli [117, 118, 119].

If little is known on their adhesive properties, the best known determinants of cariogenicity in lactobacilli are their capacity to produce acids and their ability to grow and survive in acidic environment.These bacteria have a fermentative metabolism and according to the species, two metabolic types exist; some species use homolactic fermentation and produce only lactic acid and others, using heterolactic fermentation produce lactic acid, CO_2_, acetic acid or ethanol. These latter are also able to metabolize pentose and pentitols. Whatever the metabolic method used by the lactobacilli, it results in an acidification of the environment. Since the work of Stephan, numerous studies have shown not only the acidogenic capacities of lactobacilli but also their acid tolerance. These bacteria can cause a decrease in environmental pH until values are less than 4.5 [120-124]. These species are able to survive in a pH up to 2.2 [125-127].

In order to limit this cariogenic potential, the use of sugar substitutes such as polyols has been recommended [128, 129]. Numerous studies have given contradictory results on lactobacilli‘s capacity to ferment sorbitol or xylitol. Some authors have shown that sorbitol was not fermented by lactobacilli [130] but, in other studies these bacteria were able to metabolize this polyol, often slower than sucrose, but the final pH was less than the critical value of 5.5 [124, 131-134]. Although the metabolic aspect of xylitol has been mainly studied for the oral streptococci, some lactobacilli species seem to be able to ferment this polyol [124, 131, 135, 136]. However, other contradictory studies showed that lactobacilli were unable neither to grow nor to produce acids in the presence of this molecule [122, 132, 137, 138].

These controversial studies could raise questions about the intensive use of xylitol the in prevention of decay, since some species firstly unable to metabolize it are able to adapt and to ferment this polyol [139].

### 5. Influence of Lactobacilli on Oral Health

In some cases, lactobacilli could also play a beneficial role by inhibiting the growth of some cariogenic bacteria. Michalek *et al.* [90] present evidence that instead of contributing to the caries process, *L. casei,* if present in plaque, may reduce *S. mutans*- induced dental caries in gnobiotic rats.

Ahumada *et al.* [91] have shown that 36% of lactobacilli isolated from the tongue were able to inhibit the growth of *S.mutans*. The homofermentative group produced more inhibitive substances than the heterofermentative group. In another study, the same authors show that lactobacilli isolated in patients with active caries produced more inhibitive substances and substances active on more streptococci species (*mutans, sanguis, anginosus, salivarius…*).

Sookhee *et al.*[140] isolated in the oral cavities of sound subjects two species of lactobacilli (*L. pacasei paracasei* and *L. rhamnosus*) capable of having an antimicrobial activity against streptococci (*S.mutans*, *S. sanguis, S .salivarius*), *Staphylococcus aureus*, *Actinomyces viscosus*, *Porphyromonas gingivalis* and *Candida*.

These results were confirmed by the study of Koll-Klais *et al.* [141]*,* in which 69% of tested lactobacilli inhibited *S. mutans*, 88% *Actinobacillus actinomycetemcomitans*, 82% *Porphyromonas gingivalis* and 65% *Prevotella intermedia. *The greatest antimicrobial activity was associated to *Lactobacillus paracasei*, *Lactobacillus plantarum*, *Lactobacillus rhamnosus*, and *Lactobacillus salivarius*. Strains isolated in periodontically sound patients showed the lowest antimicrobial activity against *S. mutans *than**the strains isolated in patients with chronic periodontitis. Ishikawa *et al.* [142] reported that in an *in vitro* co culture system, *L. salivarius* inhibited *Porphyromonas gingivalis*, *Prevotella intemedia* and *Prevotella nigrescens* within 24h. These authors have also carried out a clinical study showing that dayly intake of tablets containing *L.salivarius* decreased the number of anaerobic rods in saliva after 4 weeks of administration.

Another effect is the inhibition of adherence*,* a major cariogenic factor of *S. mutans*.* L. fermentum* and its culture supernatant inhibit the production of water insoluble glucan by *S. mutans*. The growth of *S.mutans* is not inhibited but its *in vitro* adherence is totally inhibited [143]. Recently, Simark-Mattsson *et al*. have demonstrated that oral lactobacilli, capable of inhibiting MS *in vitro*, occurred naturally in healthy young subjects [144].

This possible protective activity has been partially confirmed by some recent reports on probiotics effects on oral microflora. Probiotics are food ingredients with a sufficient number of viable microorganisms that are beneficial to the health of the host. They are especially known for improving intestinal microbial health and have been used extensively in fermented milk products such as yoghurt for many years. The most frequently used bacteria in these food products include lactobacilli and bifidobacteria (*Bifidobacterium bifidum*).

Some authors have investigated the role of lactobacilli as probiotics in oral health. According to Bussher *et al*. *L. acidophilus *and* L. casei* present in yoghurts can colonize the oral cavity because they are able to adhere to enamel. A one week consumption of this yoghurt caused a removal of other lactobacilli in dental plaque and in saliva. Näse *et al*. showed that long-term consumption of milk containing LGG caused a significant reduction in caries risk in day care children. 

According to Petti *et al*. the regular consumption of this yoghurt can decrease the salivary lactobacilli and *S. mutans* rate; but, as *L. bulgaricus* contained in that product do not colonize the oral cavity, its effect disappears when its intake ends. Ahola *et al*. studied whether a short-term consumption of cheese containing LGG and *Lactobacillus rhamnosus *LC 705 diminishes caries-associated salivary microbial counts in young adults. This cheese seems to reduce the risk of high counts of *S. mutans* and yeasts and so, reduces the carious risk [145-149].

On the other hand, Montalto *et al*.[150] have shown that the administration of probiotics during a 45-day period, caused an increase of the salivary rate of lactobacilli but, the streptococci rate was not significantly modified. These authors recommend a regular surveillance of oral cavities of subjects consuming robotics. In Matsumoto *et al’s* study[151], super infection with *S. mutans *MT8148R and *L. salivarius* LS1952R enhanced the establishment of MT8148R on the molar tooth surfaces, which resulted in an enhancement of dental caries induction there. As super infection with the two species did not enhance the establishment of LS1952R on the molar tooth surfaces, co aggregation did not appear to occur. The environment produced by *L. salivariuss *LS1952R may influence *S. mutans *to grow and multiply. Recently, a strain of *L. salivarius  *was reported to have a robotic action against periodontopathic bacteria and mutans streptococci owing to its ability to attach to the salivary pellicle on the tooth surface [152]. Unlike previous studies, Yli-Knuuttila *et al*.[153] have shown that a strain of *L. rhamnosus* GG was not able to colonize the oral cavity of 56 volunteers consuming a probiotic containing drink during 14 days.

Clearly, lactobacilli play a significant role within the oral ecosystem whether oral health or carious diseases.

The multiplicity of the techniques used in the various studies does not allow the emergence of one *Lactobacillus* species that could be especially implicated in dental caries. However, species belonging to the *casei* group seem to be associated with this decay.

More precise studies could give a better understanding of the pathogenic potential of these species and could allow the development of more specific caries prediction tests.

## Figures and Tables

**Table 1.** Species of Lactobacilli Identified in Saliva of Sound Subjects

Identified species	Tennpaisan and Dahlen	Smith *et al.*	Botha *et al.*	Koll-Klais *et al.*
**casei group** *L. rhamnosus*. *L. casei* *L. paracasei* *L. alactosus* *L. plantarum* *L. acidophilus* *L. cellobiosus* * L.fermentum* *L. salivarius* *L.buchneri* *L. pentosus* *L. brevis* *L. vaginalis* *L. xylosus* *L.oris*	25% 5% - NI 1% 3% - 38% 5% NI NI NI NI NI NI	- + - NI - + - + + - - ++ NI NI NI	- 29% - NI 1.8% 5.3% - - 46.3% - - 5.5% NI NI NI	18% - 36% - 36% 9% - 64% 27% - - - - - 45%
**delbrueckii group** *L. delbrueckii* * L.leichmanii* *L. gasseri* *L. crispatus*	- NI - -	+ NI - NI	- NI 1.5% NI	9% - 9% 9%

NI: non identifiable with the method used. -: non recovered.

**Table 2.** Species of Lactobacilli Identified in Saliva of Subjects with Caries

Identified species	Nancy *et al.*	Munoz-Jeldrez *et al.*
**casei goup** *L. rhamnosus*. *L. casei* *L. paracasei* *L. alactosus* *L. plantarum* *L. acidophilus* *L. cellobiosus* * L.fermentum* *L. salivarius* *L.buchneri* *L. pentosus* *L. brevis* *L. vaginalis* *L. xylosus*	++ + - NI + + - + + - - - NI NI	- - - - 4-8% 3-24% - 1-35% 5-3% - - - - 2-29%
**delbrueckii goup** *L. delbrueckii* * L.leichmanii* *L. gasseri*	+ NI -	- - -

**Table 3.** Species of Lactobacilli Identified in Dental Plaque

identified species	Carlsson* et al.*	Meiers *et al.*	Milnes and Bowden	Nancy *et al.*	Ahumada *et al.*	Schüpbach* et al.*	Botha *et al.*
**casei group** *L. rhamnosus*. *L. casei* *L. paracasei* *L. alactosus* *L. plantarum* *L. acidophilus* *L. cellobiosus* * L.fermentum* *L. salivarius* *L.buchneri* *L. pentosus* *L. brevis* *L. confusus*	22% 48% NI 11% NI 9.6% NI 9.6% NI NI NI NI -	- 77.8% - - + + - - - - - + -	- 6.2% - - 28% 9.3% - 31% 12.5% - - 12.5% -	++ + - NI + + + + + - - - NI	23.1% 7.7% 23.1% NI 30.8% - - 7.7% - - - - NI	- - - NI - - - + + - - - NI	2.4% 18.5% - 1.2% 9% 9.2% - 4.6% 36.2% - - 9% 9%
**delbrueckii group** *L. delbrueckii L.leichmanii* *L. gasseri*	NI NI NI	+ + -	- - -	+ NI -	7.7% NI -	- NI +	- NI 13.8%

**Table 4.** Species of Lactobacilli Identified in Children´s Caries

Identified species	Nancy *et al.*	Marchant *et al.*
**casei group** *L. rhamnosus* *L. casei* *L. pseudoplantarum* *L. plantarum* *L. acidophilus* *L. casei casei* *L. cellobiosus* *L.fermentum* *L. salivarius* *L.buchneri* *L. pentosus* *L. brevis*	++ - + + + + + + - - - -	24% 38% NI11% NI NI 2% 34% 19% 7% 4% 7%
**delbrueckii group** *L. delbrueckii * *L.leichmanii*	+ NI	NI NI

**Table 5.** Species of Lactobacilli Identified in Adult´s Caries

Identified species	Ozaki*et al.*	Botha *et al.*	Martin *et al.*	Buyn *et al.*	Munson *et al.*	Chhour *et al.*	Schüpbach *et al.*	Hahn *et al.*	Chavez de Paz *et al.*
**casei group** *L.casei* *L. plantarum* *L.pseudoplantarum* *L. paracasei * *L. rhamnosus,* *L. fermentum* *L. acidophilus* *L. buchneri* *L. pentosus* *L. salivarius* *L. coryneformis* *L. brevis* *L.oris* *L.reuteri* *L. graminis* *L. sake* *L. vaginalis* *L. bavaricus*	+ + N I N I N I N I N I N I N I N I N I N I N I N I N I N I N I N I	+ - N I + + + + - - - N I - N I N I + + N I +	- + N I + + + + - - N I - N I N I N I NI - N I -	+ - NI + + + - - - + NI - - - NI - - NI	+ + - - + + - + + + - - + + - - + -	+ NI NI NI + + NI NI NI + NI NI NI + NI NI NI NI	- - + - + + - - - - N I + N I N I N I - N I -	+ + NI - + + + + + - NI - NI NI NI + NI NI	+ + NI + + - + NI NI + NI NI NI - NI NI NI NI
delbrueckii group *L. hamsteri* *L. jensenii* *L. gasseri* *L. ultunensis* *L. crispatus* *L. gallinarum* *L. delbrueckii* *L. colehominis* *L.panis*	N I N I N I N I N I N I N I N I N I	+ + - N I - N I - N I N I	N I N I - N I - N I - N I N I	NI - + + + + + - -	- - + + - - + + -	NI NI + + + NI + NI +	N I N I + N I N I - N I N I NI	NI NI - NI - NI - NI NI	NI NI - NI + NI + NI NI
leuconostoc group *L. minor* *L. confusus* * *	N I N I	+ +	- -	NI NI	- -	+ NI	NI NI	NI NI	NI NI

NI: non identified.
